# Editorial for the Special Issue on “Graphene-Related Materials: Synthesis and Applications”

**DOI:** 10.3390/nano12162740

**Published:** 2022-08-10

**Authors:** Michal Otyepka, Dimitrios A. Giannakoudakis, Aristides Bakandritsos

**Affiliations:** 1 Regional Centre of Advanced Technologies and Materials, Czech Advanced Technology and Research Institute (CATRIN), Palacký University, Šlechtitelů 27, 783 71 Olomouc, Czech Republic; 2 IT4Innovations, VŠB–Technical University of Ostrava, 17. Listopadu 2172/15, 708 00 Ostrava-Poruba, Czech Republic; 3 Department of Chemistry, Aristotle University of Thessaloniki, 54124 Thessaloniki, Greece; 4 Institute of Physical Chemistry, Polish Academy of Sciences, Kasprzaka 44/52, 01-224 Warsaw, Poland; 5 Nanotechnology Centre, Centre of Energy and Environmental Technologies, VŠB–Technical University of Ostrava, 17. Listopadu 2172/15, 708 00 Ostrava-Poruba, Czech Republic

Since the groundbreaking discovery of graphene by Geim and Novoselov in 2004, there has been continuous research focused on the utilization of graphene (GR) and graphene-related materials (GRms) in technologically high-impact applications, spanning from electronics, sensing, and spintronics, to catalysis, energy storage, and environmental remediation. The one-atom-thick two-dimensional crystal of conjugated carbons arranged in a honeycomb lattice results in remarkable physicochemical properties, such as electrical and thermal conductivity, transparency to light, mechanical flexibility, and strength. Another critical feature of GR is the possibility to undergo controllable chemical doping with diverse atoms and/or functionalization with chemical groups in an out-of-plane geometry, allowing the desirable tuning of physical, chemical, magnetic, or/and optoelectronic properties for specific applications. Moreover, this broad set of properties opens the avenue for the design and development of novel and multifunctional (nano)materials via the integration of GR or GRms in composites and hybrids, further broadening the family and applicability of this exciting material.

This Special Issue, “Graphene-Related Materials: Synthesis and Applications” aimed to collect selected, original and innovative articles presenting recent trends and advances on the design, synthesis, characterization, and applications of graphene-based materials and their composites in various fields of application. In particular, seven research articles were published. Arif et al. presented a successful one-pot blended reflux condensation route for the synthesis of nitrogen-doped reduced graphene oxide nickel-cobalt (N-rGO-Ni/Co) and nickel-silver (N-rGO-Ni/Ag) nanocomposites, which presented superior and stable electrocatalytic activity for Oxygen Reduction Reaction (ORR) [1]. Sedajova and co-workers explored the synthesis of conductive Graphene Acid (GA), which is dispersible in water and can be produced on a large scale from fluorographene [2]. The lightweight supercapacitor electrodes prepared by GA revealed extremely high stability/durability. Jakubec and co-workers showed that the partially graphitic structure of natural flax-derived carbon plays a crucial role in electrochemical double-layer (EDLC) capacitors since the material presented high values of specific capacitance, high rate, and outstanding lifetime [3]. Chalmpes et al. presented the formation of carbon soot [4], consisting of nanosheets, as well as dense and hollow spheres, by the direct contact of liquid bromine with metallocene at ambient conditions. The Br-rich material exhibited visible photoluminescence (under ultraviolet irradiation) due to surface passivation linked to the amine surface groups. Placha and co-workers presented the preparation of a polymeric hybrid consisting of cationic polymer and graphene or graphene oxide [5]. The attachment of polymer onto the carbonaceous phase led to elevated antibacterial capability for the hybrids. Su and co-workers fabricated conductive cotton fabrics using “active” graphite (which was obtained after oxidation by Hummers’ method, acid chlorination, and reaction with the para-ester) [6]. Adding a small amount of “active” graphite (3% by weight) led to a durable fabric with more than 50% increased antistatic properties compared to the pristine fabric. Zhang et al. presented an environmental-friendly and practical approach for ultrasound-assisted graphene exfoliation based on Diels–Alder reaction, in which N-(4-hydroxyl phenyl) maleimide acted as the intercalation agent and as a dienophile reagent [7]. The resulting hydroxyl phenyl functionalized exfoliated graphene showed a thickness of 0.5–1.5 nm and an average lateral size in the range of 500 to 800 nm. The received nanomaterial showed a 10-fold higher removal/adsorption efficiency against a phenylpropanoid/stilbenoid (resveratrol) as compared to well-performing macroporous resins reported in the literature.

We hope this Special Issue will stimulate further developments and new ideas via fruitful discussions between experts in academia and industry working in the field of Graphene-Related Materials.
